# The impact of death and dying on the personhood of senior nurses at the National Cancer Centre Singapore (NCCS): a qualitative study

**DOI:** 10.1186/s12904-022-00974-9

**Published:** 2022-05-20

**Authors:** Chong Yao Ho, Nicole-Ann Lim, Yun Ting Ong, Alexia Sze Inn Lee, Min Chiam, Gillian Phua Li Gek, Shiva Sarraf-Yazdi, Stephen Mason, Lalit Krishna

**Affiliations:** 1 Yong Loo Lin School of Medicine, National University of Singapore NUHS Tower Block, 1E Kent Ridge Road, Level 11, Singapore City, 119228 Singapore; 2 Division of Cancer Education, National Cancer Centre Singapore, 11 Hospital Dr, Singapore City, 169610 Singapore; 3 Division of Supportive and Palliative Care, National Cancer Centre Singapore, 11 Hospital Dr, Singapore City, 169610 Singapore; 4 Lien Centre for Palliative Care, Duke-NUS Medical School, Singapore 8 College Road, Singapore City, 169857 Singapore; 5 Duke-NUS Medical School, National University of Singapore, 8 College Rd, Singapore City, 169857 Singapore; 6 Palliative Care Institute Liverpool, Academic Palliative & End of Life Care Centre, University of Liverpool, United Kingdom Cancer Research Centre, University of Liverpool, 200 London Rd, Liverpool, L3 9TA UK; 7 Centre of Biomedical Ethics, National University of Singapore 21 Lower Kent Ridge Rd, Singapore City, 119077 Singapore; 8 PalC, The Palliative Care Centre for Excellence in Research and Education, Singapore PalC c/o Dover Park Hospice, 10 Jalan Tan Tock Seng, Singapore City, 308436 Singapore

**Keywords:** Nurses, Oncology, Palliative Medicine, Personhood, Professional Identity Formation

## Abstract

**Background:**

A nurse’s role in caring for the dying is fraught with ethical, professional, and psychosocial challenges that impact how they perceive their roles as professionals. When unsupported, nurses caring for the dying experience burnout, career dissatisfaction and leave the profession. Better understanding of how caring for the dying affects the professional identity formation (PIF) of nurses will guide efforts to better support nurses.

**Methods:**

Guided by new data on the subject, we adopt the theoretical lens of the Ring Theory of Personhood (RToP) to evaluate how caring for the dying impacts the values, beliefs, principles, professional identities and personhood of nurses. We employ Krishna’s Systematic Evidence-Based Approach (SEBA) to guide the design and piloting of the semi-structured interview tool.

**Results:**

Analysis of interviews with eight senior nurses in Supportive, Palliative and Oncology care revealed three domains: Identity 1) Formation; 2) Conflict and 3) Refinement. Identity Formation occurs early in a nurse’s career, upon entering a new specialist field, and at the start of Supportive, Palliative and Oncology care. Identity Formation reveals significant changes to how self-concepts of professional identities are tied to individual concepts of personhood. Caring for the dying, however, resulted in Conflicts between values, beliefs, and principles within regnant concepts of personhood and their professional duties. These conflicts are captured as conflicts within (‘disharmony’) and/or between (‘dyssynchrony’) the rings of the RToP. These conflicts can result in changes to self-concepts of personhood and professional identities. Identity Refinement sees experience and timely support helping nurses attenuate the impact of difficult experiences. This reduces the risk of burnout and mitigates changes to their professional identities. Identity Refinement helps them develop a ‘rooted identity’ which remains relatively consistent in the face of adversity.

**Conclusions:**

Ongoing Identity Construction amongst nurses, particularly in caring for the dying, underscore the host organisation’s role in ensuring structured, longitudinal, accessible, and personalised assessments and support of nurses, especially when they are prone to dyssynchrony and disharmony whilst caring for the terminally ill. Further study into assessment methods and the role of the environment is critical.

**Supplementary Information:**

The online version contains supplementary material available at 10.1186/s12904-022-00974-9.

## Introduction

Caring for the dying is often fraught with ethical, professional, and psychosocial challenges for healthcare professionals (henceforth HCP)s [1–4]. A number of studies have also suggested that when unsupported, reflections, meaning-making and integration of these experiences may result in maladaptive coping mechanisms, burnout, attrition in the profession and compromised patient care [5–7].

Kuek, Ngiam [8] notes that poorly processed experiences impact self-concepts of professional identity and personhood and have significant implications on a nurse’s personal and professional choices [9–11]. The authors further suggest that the impact of caring for the dying also affects evolving concepts of personhood and PIF. This may be captured through the lens of the Ring Theory of Personhood (RToP) [12, 13]. The authors posit that understanding self-concepts of personhood and PIF could better facilitate support of healthcare professionals. Yet given the different roles and experiences of the different specialities, changing concepts of personhood may be speciality specific [14, 15].

Guided by these findings, a prospective study of the experiences of senior nurses in Palliative, Supportive and Oncology Care is proposed using the Ring Theory of Personhood (RToP).

### The ring theory of personhood (RToP)

**Fig. 1:**
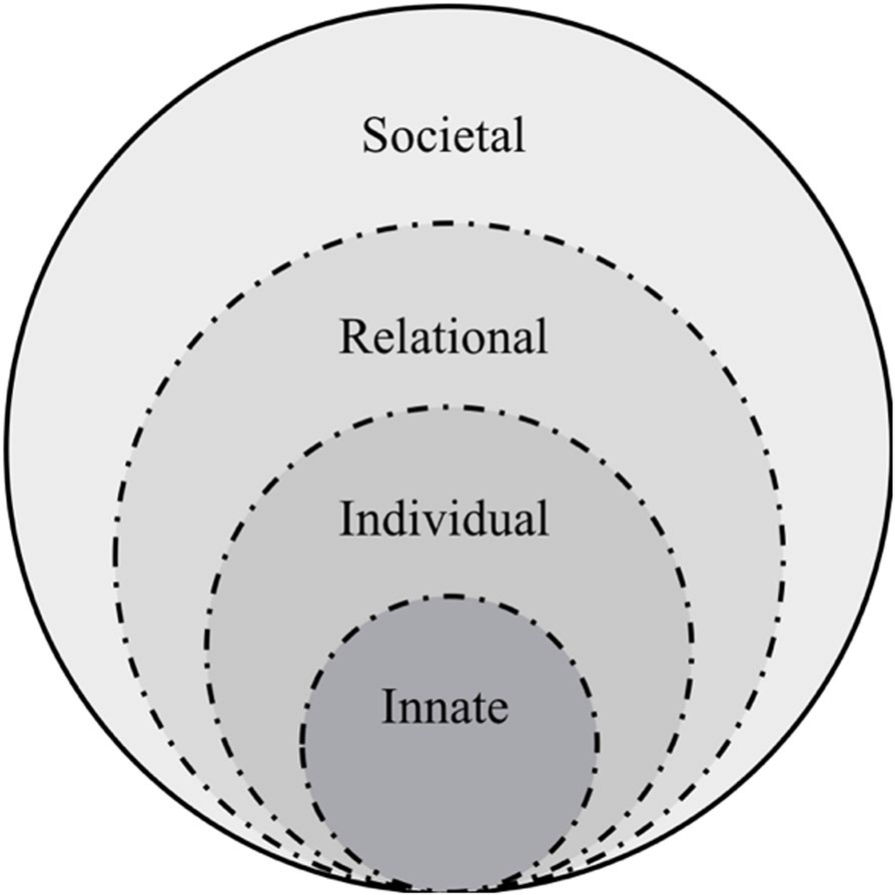
The Ring Theory of Personhood

The RToP suggests that personhood is composed of the Innate, Individual, Relational and Societal domains (Fig. 1). Each domain contains specific beliefs, moral values, ethical principles, familial mores, cultural norms, attitudes, thoughts, decisional preferences, roles and responsibilities that create specific identities. The Innate Identity considers religious, gender, cultural, community-based beliefs, moral values and ethical principles [16–18]. The Individual Identity encompasses personal values, beliefs, personalities whilst the Relational and Societal Identities pivot on familial and societal values, beliefs, expectations, and principles, respectively [19–22].

Balancing the sometimes competing professional, sociocultural, legal, ethical, and personal considerations, roles and responsibilities associated with these identities can, however, result in dissonance. We discuss the issue of dissonance a little later.

## Material and Methods

### Systematic evidence based approach (SEBA)

Krishna’s Systematic Evidence-Based Approach (SEBA) is employed to guide the design and piloting of the semi-structured interview tool. SEBA’s constructivist approach [23–29] and relativist lens [30–34] acknowledges the notion that identity and personhood are sociocultural constructs governed by individual values, beliefs, principles, personal narratives and contextual considerations.

Each stage of the adapted SEBA approach involves input from an expert team consisting of a medical librarian from the Yong Loo Lin School of Medicine (YLLSoM) at the National University of Singapore (NUS), and local educational experts and clinicians at the National Cancer Centre Singapore (NCCS), the Palliative Care Institute Liverpool, YLLSoM and Duke-NUS Medical School (henceforth the expert team). The expert team ensures that the SEBA methodology is employed in a consistent manner, and enhances the transparency and accountability of the research process.

**Fig. 2:**
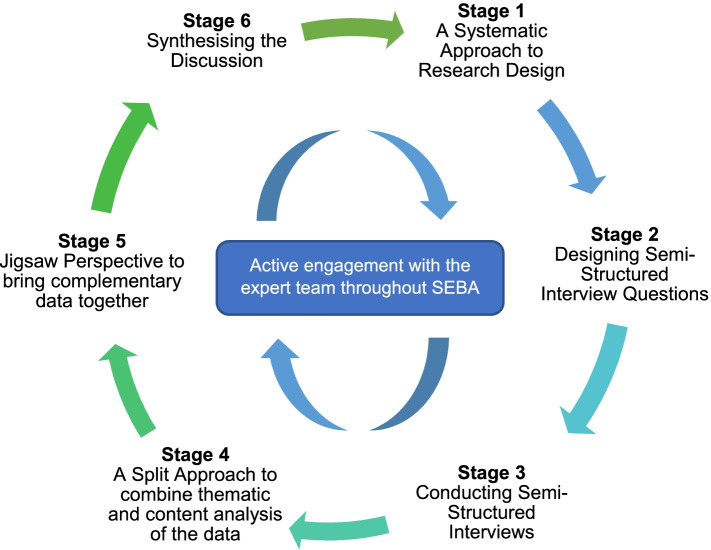
The Prospective SEBA Process

The adapted SEBA methodology consists of the following six stages: 1) Systematic Approach, 2) Designing the semi-structured interviews, 3) Conducting the interviews, 4) Split Approach 5) Jigsaw Perspective, and 6) Synthesis of the Discussion (Fig. 2).

### Stage 1. Systematic approach

Guided by the current data the expert and research teams adopted a systematic approach to review the key findings of reviews on the impact of caring for the dying on nurses to design a semi-structured interview questionnaire. The design of the questionnaire was shaped by the Ring Theory of Personhood (RToP). The goal of this qualitative study was to identify and understand how senior nurses in a quaternary cancer center in Singapore cope with caring for dying patients. The data is anticipated to better support the individual development and needs of senior nurses in Palliative Care teams. The findings may even be useful to senior nurses in other settings and could inform efforts to study similar experiences amongst physicians and medical students.

Ethics approval (2021/2176) was obtained from the SingHealth Combined Institutional Review Board.

### Stage 2. Semi-structured interview design

Drawing upon the findings of the systematic analysis of current data in Stage 1, the research and expert teams designed semi-structured interview questions aimed at facilitating an in-depth exploration into the lived experiences of senior nurses caring for dying patients and the impact of these experiences on their personal beliefs, values, principles and practices [35]. The interview guide was then reviewed by local Palliative Care experts and qualitative researchers as part of an informal Modified Delphi Process. The interview guide is included in Appendix 1.

### Stage 3. Conducting semi-structured interviews

We conducted audio-recorded semi-structured interviews with senior palliative care and oncology nurses at the National Cancer Centre Singapore (NCCS), a tertiary cancer specialist centre, between July to August 2021 via video conferencing.

Eligible participants included Advanced Practice Nurses (APN)s in the Division of Supportive and Palliative Care (DSPC) and the Division of Medical Oncology. APNs are nurses who were selected for their experience and abilities to complete a master’s degree in nursing. APNs in the Supportive and Palliative Care teams rotate between Supportive Care and Palliative Care in outpatients and inpatient settings. In Supportive Care, APNs provide care to symptomatic patients in the early and curative stages of their illness. They also provide support to cancer survivors and participate in clinical research. Much of this involves telephone consults and clinical work. Palliative Care involvement occurs within the inpatient consult team at the Singapore General Hospital and involves providing Palliative Care support to terminally ill patients. These patients may be in the final stages of their oncology, haematological, renal, cardiac, or respiratory conditions. As part of the consult service, APNs in the Palliative Care teams often function independently, providing advice to the primary care team, participating in multidisciplinary team meetings, advising on care determinations and representing the Palliative Care team at family meetings.

Purposive sampling was conducted with email invitations sent to all APNs involved in the care of patients at the end of life. The emails contained participant information sheets, study information, and details on the nature, duration and aims of the audio-recorded interviews. The e-mail invitations stressed participant anonymity and highlighted the participant’s right to withdraw from the study at any point and without prejudice.

Of the 13 senior nurses at NCCS, three were in administrative posts and one was posted to the chemotherapy suites. Eight of nine eligible nurses participated. Two trained members of the research team (ASIL and MC) sought verbal and written consent before conducting the interviews. Each interview lasted approximately 45 min and data saturation was reached at the seventh interview with no new themes identified. Audio recordings were transcribed verbatim using the NVivo 12 Software, anonymised and their integrity verified by the participants. The research team adopted an inductive approach to analysing the ‘member checked’ interview transcripts.

### Stage 4: Split approach

Concurrent use of Hsieh and Shannon [36]’s approach to directed content analysis and Braun and Clarke [37]’s thematic analysis ensured a comprehensive, reproducible, transparent analysis [38–40].

#### Thematic analysis

Braun and Clarke’s approach to thematic analysis was adopted to analyse the data in acknowledgement of personhood and PIF’s socioculturally influenced roots. In addition, thematic analysis also engages qualitative and quantitative knowledge synthesis, as well as descriptive and observational studies that do not lend themselves to statistical pooling and analysis [41–48].

#### Coding framework

The research team independently constructed ‘codes’ from the ‘surface’ meaning of the text from a randomly selected sample of the same 10 articles. Three members of the research team discussed the ‘codes’ online and categorised them into corresponding groups. Following discussion with senior members of the research team, the research team employed ‘Sandelowski and Barroso [49]’s approach to ‘negotiated consensual validation’ to determine the final list of titles to be reviewed. The research team agreed upon a coding framework and code book using ‘negotiated consensual validation’.

Using the coding framework and code book, the members of the research team independently coded and analysed the remaining articles. Subthemes and themes were developed and agreed upon at online discussions using ‘negotiated consensual validation’.

#### Directed content analysis

Concurrently, three other members of the research team not involved in the thematic analysis of the transcripts, employed Hsieh and Shannon’s approach to directed content analysis to independently analyse the included articles. Directed content analysis serves to address the apparent failure of thematic analysis to consider contradictory data [50].

Directed content analysis use of pre-determined categories drawn from ‘key’ publications also serves to draw attention to specific features of interests, and account for inherent biases and differing interpretations of terminologies. Here, Kuek, Ngiam [8]’s systematic scoping review entitled “*The impact of caring for dying patients in intensive care units on a physician's personhood: a systematic scoping review*” and Thompson, Shura [51]’s *“"Doing" healthcare at end-of-life: Identity tensions, negotiations, and conflicts”,* were adopted as they represented the most recent reviews in the field and together best captured the key concepts of personhood and professional identity formation amongst healthcare practitioners in the end-of-life care setting. Three independent members of the research team discussed the categories at online meetings and ‘negotiated consensual validation’ was employed to determine the final list of categories to enhance the trustworthiness of the coding process [52].

### Stage 5 of SEBA: Jigsaw perspective

The Jigsaw Perspective brings overlapping elements of the themes and categories together to create a holistic perspective of PIF. To do so, Phases 4 to 6 of France and colleagues’ adaptation of Noblit and Hare [53]’s seven phases of meta-ethnography is employed. This process sees the themes, categories along with their subcategories and subthemes reviewed [54, 55]. *Reciprocal translation* was employed to determine if the themes and categories could be used interchangeably. The combined themes and categories are referred to as domains.

## Results

All eight APNs were female and attached to the Palliative Care team at DSPC with 8 to 22 years of nursing experience. Seven were senior palliative care nurses and one was from medical oncology on a one-year posting in Palliative Care. All were involved in the care of dying patients with two in the inpatient setting and six in the outpatient setting (Appendix 2). The themes identified were Identity Formation, Identity Conflict, and Identity Negotiation. The categories were the Innate, Individual, Relational and Societal Rings, Identity Formation, Dyssynchrony, Disharmony, and Identity Negotiation. The Jigsaw Perspective revealed three final domains: 1) Identity Formation; 2) Identity Conflict and 3) Identity Refinement.

Follow up clarifications on the transcripts following member checking did not reveal new data.

### Domain 1: Identity formation

It was found that early in their nursing careers, upon entering a new specialist field, and at the start of their Palliative Care careers, nurses experienced significant changes to how they conceived their personhood and identities. These changes are captured in each of the four rings of the RToP as described below.

#### The societal ring

Commencing a new role highlighted gaps in knowledge, and skills (N1, N2, N7, N8) and limitations in coping mechanisms (N2, N4, N6, N7). This was evident on starting the APN role:

“*When I joined, I actually wanted to quit...because it's emotionally draining .. so many cases.... But along the way, ..I begin to learn from them…”* (N6).

These issues were addressed through role modelling (N2, N4, N8) and shared experiences (N2, N5, N8) within the team. This helped nurses transition from actively managing patients with curative intent, to caring for the dying. This improved their communication skills, clinical interactions (N2) and boosted their patients’ treatment adherence (N2, N5, N8).

Two nurses noted that improvements in their ability to empathise allowed for more personalised, dignity-conserving care (N3, N5).

*“[Compared to when I was a junior nurse], I think with more experience, I realised the need to talk to patients in different ways and understand their point of view… [patient care] is very individualised*” (N5).

#### The relational ring

Caring for dying patients impacted the nurses’ attitudes toward their own families. For five nurses, (N3, N4, N6, N7, N8) experiences with dying patients created distance between them and family members family – *“Actually, my parents, my mom especially, she ever made comments like I treat patients better than I treat her” (N3).*

Nurses (N3, N4, N6, N7, N8) also reported a growing distance due to their family’s inability to fully appreciate the demands of their work. Nurse (N2) reported that she “*used to tell [her] family certain interesting stories but then realised sometimes it's a little bit difficult for them to understand… because they're not medically trained*”. For these nurses, this shift left them feeling inadequately supported by their loved ones.

#### The individual ring

Shifts in personal practices brought on by increased awareness and greater experience in Palliative Care featured strongly in the Individual Ring of all eight nurses. Nurse (N2) reported a greater sense of self-awareness stating, *“sometimes [I didn’t] realise that [I was] already very affected…[I] start behaving differently from how [I was]”.*

Greater experience brought with it shifts in personal philosophies. Nurse (N1) stated – “*It just helps me to learn that time is a present… You don’t know if tomorrow you're going to wake up or not.” (N1).*

#### The innate ring

All eight nurses believed that their experiences in Palliative Care engendered contemplation of their own mortality. For some, this mitigated their fear of death (N1, N3) or strengthened their religious beliefs (N1, N6, N8). It also helped crystalise their own life choices – “*I told my husband… if anything happen[s] to me… don't resuscitate me…let me go*”. (N6).

### Domain 2: Identity conflict and its influences

Caring for the dying resulted in conflicts between values, beliefs, and principles within the rings of the RToP (‘disharmony’) and between the rings (‘dyssynchrony’). We summarise examples of disharmony and dyssynchrony and the factors that ameliorate or exacerbate them, in Table 1.

**Table 1:** Types of identity conflict defined as disharmony and dyssynchrony, and their modulating influences

Identity Conflict		*Quotes*	Protective Influences	Exacerbating Influences
**Disharmony** *within* **Ring**	Professional Expectations vs. Work Obligations	*“I wish I had time to explore my patients emotional or psychosocial needs…but it's not always possible….”* (N3)	Enhanced **professional skills** (N1, N2, N4) better prepared nurses for difficult patient encounters Nurses received emotional support *and* practical advice through **informal support from nursing and medical colleagues** (N8) and through **formal support programs** (N6) An **open and supportive non-hierarchical work environment enhanced teamwork, self-care and working practice**. (N4, N6, N8)	**Heavy workloads** and **responsibilities** (N7, N8) **Lack of protected out-of-hospital time** for self-care (N7, N8) **Lack of opportunities to grieve** (N6) and **for support** (N7) (e.g., mortality rounds)
	Professional Role vs. Professional Hierarchy	*“There is still hierarchy..without seniority, some people they don’t accept your instructions.”* (N1)		
**Dyssynchrony** *across* Rings	Innate vs. Societal	*“A patient asked me to pray for her…not being in that religion, I won't be able to. And the patient can get a bit offended.”* (N4)		
	Individual vs. Societal	*“Patients who are similar in age and background [to me]…are challenging because it reflects [my] own mortality.”* (N1)		

### Domain 3: Identity refinement

Addressing conflicts within and between the rings is key to developing a relatively consistent identity even in the face of ‘disharmony’ and/or ‘dyssynchrony’.

#### Societal ring

In learning to cope with the nature of Palliative Care, nurse (N1) acknowledged a shift in her thinking. Likening healthcare professionals to gardeners, she recognised her own limitations: *"Life has four seasons. The doctors are just like the gardeners who look after the flowers in the different seasons, but the gardener cannot change the seasons. [For] some of them, the winter just comes a bit too early”*. This allowed her to make sense of these experiences.

More reflective practice and support within the team fostered insights and changes to their outlook on life. For Nurse (N4), it resulted in her confronting her avoidance of discussions of death and dying: “*when I was a junior nurse, I avoided [challenging conversations about death and dying] … I would just go, ‘oh, let's talk about something else*”.

Nurse (N6) acknowledged her emotional entanglement and recognised a need to mourn the loss of her patients – “*we can get affected by our patients who are really close to us and sometimes we need to grieve it out*”. In addition, Nurse (N2) appreciated the importance of self-care – “*I used to [feel burnt out] … so I learnt to deal with things that I can control and let go of the things I can't control.”*

#### The relational ring

Six nurses reported that their Palliative Care experiences impacted their familial relationships (N2, N3, N4, N5, N7, N8) and parenting approach (N5, N6). It also better prepared them, emotionally, and logistically, for the death of their own loved ones (N4, N6).

#### The individual ring

For some nurses, a consistent identity came with deeper reflections, and greater prioritisation of self-care. Nurse (N7) turned her attention to improving her work-life balance by “*devot[ing] Saturdays to the things I like and spending time with the people I enjoy being with*” whilst Nurse (N6) shared how she grew to develop satisfaction, fulfilment, and happiness “*from helping people achieve a good death*”.

#### The innate ring

Over time, all eight believed that their experiences in Palliative Care changed their perception on what constituted good life and a good death. Nurse (N2) noted that a good death meant “*fulfilling my duties as nurse, daughter, a wife, as a mother. If I've done all that I think… is a good death*”. In turn, three nurses (N1, N6, N8) believed that a good death was dying in their sleep, symptom-free.

#### Addressing dyssynchrony/ disharmony

When addressing disharmony and/or dyssynchrony, nurses drew upon their existing identity to negotiate conflicts. Nurse (N1) relied on her religious beliefs and concluded that her Chinese heritage led her to believe that suffering was “*destined since [birth]*”. For Nurse (N2), incongruence between her religious views on gambling and her patient’s last wish to go to the casino required an adaptation of her beliefs – *“although it is a sin if I do it, I am just doing my job, and that's not a sin".*

Three nurses (N1, N2, N6) indicated a better appreciation of their pivotal role within the Palliative Care team and acknowledged that their insights helped the team contend with challenging situations. Nurse (N6) recognised that the anger of others did not reflect her professional capabilities but rather noted that “*…when people are angry, it's not because they're angry with you, but it's [because] of what has happened to them.”*

Nurse (N6) further added that she began to achieve a sense of closure following the demise of her patients, sharing *“I do say goodbye…I'll just tell them, you know, "thank you, thank you for allowing me to care for you”.*

Nurses (N2, N3, N5, N6, N8) also recognised the need *“to be more receptive”* to feedback (N6), and the need to address fatigue to “produce quality work” (N8).

## Discussion

### Stage 6. Synthesis of discussion

This qualitative study reveals that APNs in Supportive, Palliative and Oncology Care experience three distinct identity phases over the course of their professional development.

**Fig. 3:**
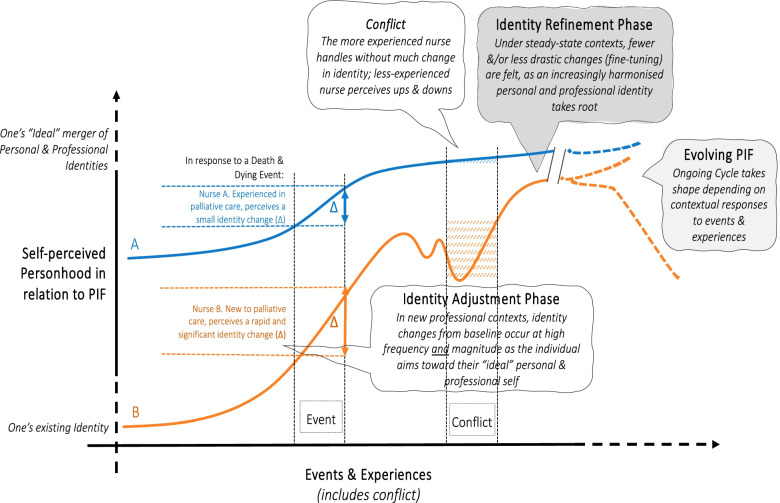
Context-specific conceptual model of self-perceived personhood in relation to an evolving professional identity formation

The Identity Formation Phase marks the start of a new stage of their professional development. This is especially apparent when nurses describe taking on a new role, accepting the responsibilities of an APN and embracing the work culture and practice of Palliative Medicine (Fig. 3). This phase is marked by experiential learning. Here, an accommodating and nurturing work environment and the presence of helpful peers and role models is especially important as nurses build knowledge and skills, experience shifts in attitudes and perspectives and grapple with uncertainty. Familial support during this stage is key.

The Identity Adjustment Phase is marked by reflection and adjustments. Adaptations that follow precipitate a re-evaluation of values, beliefs, and principles. This leads to disharmony within, and dyssynchrony between, the rings of the RToP and the need to balance and resolve these conflicts. Attending to these conflicts highlights the importance of peer and mentor support, role modelling, feedback and personalised longitudinal and appropriate guidance is clear. Attending to disharmony and dyssynchrony also relies on personal fortitude, insight, self-awareness, reflections, and the ability to adapt. Here, experience balancing competing professional obligations with personal values, beliefs, and principles; experience attending to ethically and morally complex care determinations; and experience navigating the medical structure and harnessing support from colleagues are crucial.

The process of adjustment sees changes in how APNs describe their duties as nurses, in the way they interact with family members and in their development of a greater sense of purpose and appreciation of their life experiences. Yet, these changes also see a re-balancing of priorities that may lead to readjustments in familial relationships.

The Identity Refinement Phase then see goals revisited and priorities rebalanced. This balance paves the way for the development of a more refined and consistent sense of personhood that is better able to address conflicts without significant changes to prevailing self-identities. In the Identity Refinement Phase, nurses adapt to *“threats to one’s professional identity”,* especially ones that have *“repercussions [on] their overall sense of morality and connection to society”*, by *“tactic[s] of negotiation”* [51] (Fig. 3). The notion of Identity Refinement echo Sedikides, Gaertner [56]’s Boomerang Model where a return *“to the individual self for refuelling”* or baseline concept of personhood occurs. For example, Nurse (N1) characterises this process in turning to her cultural beliefs when unable to explain the harsh realities of a patient’s deterioration to support meaning-making and maintain her professional identity.

These three phases are not discrete and show some overlap. This is in keeping with the notion that development of professional identity is an evolving, adaptive, contextual, and personalised process influenced by a variety of sociocultural and individualised considerations. These findings underline the need for an individualised, longitudinal, holistic, appropriate, and timely support as nurses address episodes of disharmony and dyssynchrony. Here, training programs to help nurses recognise conflicts both in themselves and in others, identify available support networks and build resilience are key. There ought to be accessible and robust support, assessment, feedback, and remediation systems; and nurses must have access to effective mentoring, guided reflections, personalised feedback, and role modelling. These considerations underline the import of the host organisation in overseeing the program and nurturing a conducive work environment that encourages mentorship and open communication, peer and team support, dedicated time to balance personal and professional duties, and socioculturally sensitive support strategies.

### Limitations

Whilst the SEBA methodology is well evidenced, the adaptations proposed are unique and unproven. This and the limited number of participants has curbed the analysis of the concepts of IC and IR. Whilst thematic saturation appears to be attained, the small number and limited depth of the interviews restricted effective evaluation of the environment and the impact of peer and team support. The small numbers of interviews at a single healthcare cluster in Singapore may limit the applicability of these findings outside a Southeast Asian context. The concepts of burnout though widely studied and used in the local practice, were not clarified during the interviews or during the clarifications sought from the participants.

## Conclusions

Nurses caring for the dying undergo significant changes to their thinking, beliefs, values, experiences, roles and/or codes of conduct that reflect changes to self-perceptions of personhood and professional identities. These changes are captured by the RToP. This opens the door for the possibility of adapting the RToP to evaluate changing concepts of PIF, personhood and identity and guide personalised, holistic, longitudinal and timely support of nurses in Palliative Care and indeed nurses further afield. Such a tool would be especially useful for the support of junior nurses. Further studies into how disharmony and dyssynchrony is addressed and its effects upon PIF and personhood should follow. Another focus for future research is understanding the impact of the learning and working environment and the role of the institution in supporting nurses and their well-being.

## Supplementary Information

**Additional file 1.** 

## Data Availability

All data generated or analysed during this review are included in this published article [and its appendices].
