# Supplementary material for: The impact of death and dying on the personhood of senior nurses at the National Cancer Centre Singapore (NCCS): a qualitative study
Source: BMC Palliat Care. 2022 May 20;21:83. doi: 10.1186/s12904-022-00974-9 (PMC9121572; doi:10.1186/s12904-022-00974-9)
Supplement: Supplementary file 1 — Additional file 1. [file 12904_2022_974_MOESM1_ESM.docx]

**Appendices**

Appendix 1: Interview Guide

Appendix 2: Demographic Data of Participants

Appendix 1: Interview Guide

| Questions |
| --- |
| 1. Could you share with us a case which was memorable to you or a case which changed your perspectives, beliefs, or faith? 2. Why was this case memorable to you? 3. How has this case impacted you? OR 4. How have you adapted or changed following this case? |
| 1. In the case you have highlighted, was there some conflict with what you believed in vs. what was expected of you? 2. How did this conflict affect you? 3. How did you address and cope with this conflict? |
| 1. How do you view life and death? 2. Have you always held this view or has this concept of life and death changed as you cared for dying patients? |
| 1. How do you view your relationships with people who matter to you? 2. Has this always been the case or has this changed while caring for dying patients? |
| 1. How has caring for dying patients changed the way you think about your roles and responsibilities as a nurse? 2. How does caring for dying patients affect the way you think about your patients and their family members? |
| 1. What are the skills you possess that you believe are important to have in caring for dying patients? 2. Have these been shaped by your experiences? |

Appendix 2: Demographic Data of Participants

| **Respondent**  **Nurse** | **Years of Experience** | **Speciality** | **Duties** |
| --- | --- | --- | --- |
| **1** | 8 | Division of Supportive and Palliative Care (DSPC) | Inpatient |
| **2** | 11 |  |  |
| **3** | 8 | Division of Medical Oncology (DMO) | Largely outpatient |
| **4** | 12 | DSPC | Inpatient |
| **5** | 20 |  | Outpatient |
| **6** | 20 years in nursing  9 years in palliative care |  |  |
| **7** | 10 |  |  |
| **8** | 22 |  |  |
